# MAIT Cells Display a Specific Response to Type 1 IFN Underlying the Adjuvant Effect of TLR7/8 Ligands

**DOI:** 10.3389/fimmu.2020.02097

**Published:** 2020-09-08

**Authors:** Marion Pavlovic, Christelle Gross, Chahinaize Chili, Thomas Secher, Emmanuel Treiner

**Affiliations:** ^1^INSERM UMR 1043, Centre de Physiopathologie de Toulouse-Purpan, Toulouse, France; ^2^Paul Sabatier University Toulouse III, Toulouse, France; ^3^Laboratory of Immunology, Toulouse University Hospital, Toulouse, France

**Keywords:** MAIT cells, type 1 interferons, co-stimulation, cytokines, vaccines

## Abstract

Mucosal-associated invariant T (MAIT) cells constitute a highly conserved subset of effector T cells with innate-like recognition of a wide array of bacteria and fungi in humans. Harnessing the potential of these cells could represent a major advance as a new immunotherapy approach to fight difficult-to-treat bacterial infections. However, despite recent advances in the design of potent agonistic ligands for MAIT cells, it has become increasingly evident that adjuvants are required to elicit potent antimicrobial effector functions by these cells, such as IFNγ production and cytotoxicity. Indeed, TCR triggering alone elicits mostly barrier repair functions in MAIT cells, whereas an inflammatory milieu is required to drive the antibacterial functions. Cytokines such as IL-7, IL-12 and IL-18, IL-15 or more recently type 1 IFN all display an apparently similar ability to synergize with TCR stimulation to induce IFNγ production and/or cytotoxic functions *in vitro*, but their mechanisms of action are not well established. Herein, we show that MAIT cells feature a build-in mechanism to respond to IFNα. We confirm that IFNα acts directly and specifically on MAIT cells and synergizes with TCR/CD3 triggering to induce maximum cytokine production and cytotoxic functions. We provide evidences suggesting that the preferential activation of the Stat4 pathway is involved in the high sensitivity of MAIT cells to IFNα stimulation. Finally, gene expression data confirm the specific responsiveness of MAIT cells to IFNα and pinpoints specific pathways that could be the target of this cytokine. Altogether, these data highlight the potential of IFNα-inducing adjuvants to maximize MAIT cells responsiveness to purified ligands in order to induce potent anti-infectious responses.

## Introduction

Mucosal-associated invariant T cells (MAIT) represent one of the largest subsets of innate T cells in humans, making up on average 3% of circulating T cells in healthy subjects (1, 2). Their semi-invariant TCR repertoire is hard-wired to bind conserved microbial ligands associated with the monomorphic MHC-related 1 (MR1) molecule (3–7). The most potent ligands identified thus far are pyrimidines produced from the vitamin B2 (or riboflavin) derivatives, such as 5-OP-RU (8, 9). A large number of yeasts and bacteria species are riboflavin producers and therefore synthetize these ligands, making them targets for MAIT cells (10, 11). Upon activation, MAIT cells quickly gain the ability to kill infected targets in a perforin- and granzyme/granulysin-dependent manner, as well as to produce inflammatory cytokines such as IFNγ, TNFα, GM-CSF, and IL-17 (1, 12–15). Although the network of cells interacting with, or influenced by, MAIT cells remains to be detailed, they probably also exert indirect protective functions by increasing dendritic cell (DC) maturation and B cell activation and by recruiting other immune effectors (16–18). The “innateness” of MAIT cells (19) relies both on a specific intra-thymic differentiation program, leading to acquisition of a memory phenotype and expression of specific transcription factors associated with effector functions (such as PLZF), as well as with a gradual peripheral expansion and seeding into anatomical locations directly (gut and lung mucosae) or indirectly (liver) interfacing with the environment (20–29). Importantly, MAIT cell activation by pathogens has been shown *in vivo* in human infections and could be linked to disease outcome in some conditions (30–35). Thus, MAIT cells act as innate-like T cells, recognizing highly conserved, broadly expressed microbial ligands at primary sites of pathogen infection and dissemination, and display both direct antimicrobial functions as well as the ability to influence subsequent innate and adaptive responses. The observation that all human subjects analyzed thus far dedicate a significant proportion of their T cell compartment to this specific microbial metabolite recognition system in a MHC-unrestricted manner has prompted a major interest in their potential use as targets of immune intervention in major, life-threatening infectious diseases such as tuberculosis (36).

Virtually all circulating MAIT cells display an effector-memory (CD45RA^−^CCR7^−^) phenotype and, as such, display rapid effector functions upon TCR activation. However, in contrast with other T cells with similar phenotypes, their response is blunted both *in vitro* and *in vivo* (37, 38). TCR triggering with anti-CD3 mAbs or MR1 ligands is not sufficient to induce significant IFNγ production and cytotoxic functions (39). In fact, in contrast with conventional memory CD8 T cells, resting MAIT cells express low levels of perforin and almost few granzymes, with the exception of granzyme A (5, 12, 15, 40). In contrast, activation of MAIT cells with bacteria induces full effector functions, suggesting that TLR ligands and their downstream signaling are crucial for MAIT cell activation (12, 41). Indeed, co-administration of 5-OP-RU with TLR ligands is necessary to activate and recruit MAIT cells in mice (37, 42). In humans, TLR8 ligands have been identified as potent co-activators of MAIT cells through the release of IL-12 and IL-18 by TLR-activated monocytes (43). Several laboratories have shown the potency of IL-12 + IL-18 as MAIT cells co-stimulators, but other cytokines may have similar effects, such as IL-7 (15, 39, 44–47). It is likely that these requirements for co-stimulation are the result of some kind of tolerogenic process to avoid overt stimulation of MAIT cells by the microbiota-derived metabolites in the absence of danger (48–50). Nevertheless, this is an issue when considering the prospect of immune intervention targeting MAIT cells for protection. Further, a thorough description of the cellular and molecular requirement for potent MAIT cell activation is also important to our understanding of their contribution to natural immunity against pathogens, especially for microorganisms able to evade the immune system, such as *Mycobacterium tuberculosis*.

In this report, we reassessed *in vitro* the human MAIT cells response to TLR7/8 ligands. We show that type 1 IFN play a major role in the co-stimulation of MAIT cells and provide strong evidences that these cells display a specific signaling and transcriptional program upon IFNα stimulation.

## Materials and Methods

### Blood Samples

Blood samples were obtained from buffy coats of healthy donors under an agreement with the Etablissement Français du Sang (EFS)—Midi-Pyrénées, in accordance with the EFS ethical guidelines. PBMC were isolated after centrifugation in a density gradient (Pancol, PAN Biotech) and frozen in DMSO before use. Experiments were performed after thawing except for phospho-flow and microarray analyses where fresh cells were used.

### Ethics Statement

Blood samples from anonymous healthy donors were obtained from Etablissement Français du Sang (EFS, the French National Blood Agency). Sample use for scientific purposes was carried out in accordance with convention between EFS and Centre de Physiopathologie Toulouse-Purpan. According to French law, no agreement from a local ethic committee was required.

### Cell Stimulations

Cell stimulations were performed in RPMI 1640 supplemented with antibiotics and 10% FCS. PBMC were plated at 5 × 10^6^ cells/ml in tissue culture-treated 96-well plates. R848 (10 μg/ml), gardiquimod (1 μg/ml) (both from Invivogen), IFNα2b (1000 IU/ml; Schering-Plough), IL-12 (100 ng/ml; Peprotech), and IL-18 (100 ng/ml; Peprotech) were added to the cells for 3 h before addition of OKT3 (10 ng/ml; Muromonab, Janssen-Cilag). For blocking experiments, anti-IFNαβR chain 2 (Merck Millipore), anti-IL-12 (BD Biosciences), anti-IL-18 (RD systems) or an isotype control (RD systems) was incubated with the cells 1 h before any stimulation. For the detection of intracellular cytokines, 3 μg/ml of Brefeldin A (Thermo Fisher Scientific) was added 1 h after OKT3. After 16 h of incubation, cells were harvested and processed for flow cytometry. For phosphoflow and imaging cytometry experiments, cells were incubated with 10^4^ IU/ml of IFNα2b for 15 min before processing.

### Cell Subset Isolation

For QRT-PCR experiments and functional studies, PBMC were stained with anti-TCRVα7.2-PE (Miltenyi Biotec), washed, and incubated with anti-PE microbeads (Miltenyi Biotec). After washing, cells were processed for positive selection with the autoMacs Pro Separator (Miltenyi Biotec) and the positive TCRVα7.2 fraction was collected. These cells were further stained with anti-CD5, anti-CD8, anti-CD45RA, and anti-CD161, and the CD5^+^CD8^+^TCRVα7.2^+^CD45RA^–^CD161^+^ MAIT cells and the CD5^+^CD8^+^TCRVα7.2^+^CD45RA^−^CD161^−^ conventional memory CD8 were Facs-Sorted. For microarray analysis, untouched CD8 T cells from 4 healthy donors were purified from fresh PBMC with the CD8 T cell Isolation Kit (Miltenyi Biotec), according to the manufacturer’s instructions. The purified fractions were stained with anti-TCR Vα7.2, CD45RA and CD161, and the TCRVα7.2^+^CD45RA^−^CD161^+^ MAIT cells and the TCRVα7.2^−^CD45RA^−^CD161^−^ memory CD8 T cell fractions were electronically sorted to a purity >98%. Cell sorting was performed with a FacsAria SORP equipped with four lasers (488 nm, 633 nm, 405 nm, and 375 nm) (Becton Dickinson).

### Flow Cytometry

Extracellular stainings were performed by incubating cells with the appropriate concentration of antibodies for 15 min at + 4°C, washing, and resuspending cells in PBS with 1% FCS. For MAIT cell identification, we chose to use the CD5 (sometimes together with CD2) marker as a mean to avoid artifacts resulting in CD3 downregulation induced by OKT3 stimulation. Intracellular stainings for granzyme B, perforin, and cytokines were performed after extracellular stainings and fixation and permeabilization with the Cytofix/Cytoperm (BD Biosciences), by incubating cells for 1 h with the appropriate concentrations of antibodies. For phosphoflow analyses, fresh cells were first stained with anti-CD161, washed, fixed with Max Buffer Phosflow (BD Biosciences), and permeabilized with Perm Buffer III (BD Biosciences). Cells were then stained with anti-CD8, anti-CD45RA, and either an anti-pSTAT4 or IgG2a isotype control for 1 h, before washing and analysis. The following antibodies were used: anti-TCRVα7.2 PE, anti-TCRVα7.2 FITC, anti-TCRVα7.2 PeCy7 (clone 3C10, Biolegend), anti-IFNγ FITC (clone 45-15), anti-CD8 VioBlue (clone B135/80), anti-CD107a FITC, anti-CD107a PE (clone H4A3), anti-CD8 PE (clone BW135/80), anti-CD45RA Viogreen, anti-CD45RA PE Vio770 (clone REA562), anti-CCL4 PE, anti-CCL4 APC (REA511), anti-CD161 PE Vio770, anti-CD161 APC (clone 191B8), anti-Granzyme B PE (clone REA226), anti-CD5 APC Vio770, anti-CD5 VioBlue (clone UCHT2), anti-CD2 Percp Vio700 (clone LT2), anti-TNFα FITC, anti-TNFα PE (clone cA2) (all from Miltenyi Biotec), anti-STAT1 (pY701) eFluor450 (clone KIKSI0803, eBiosciences), IgG1 isotype control eFluor450 (P3.3.2.8.1, eBiosciences), anti-STAT4 (clone 38/p-Stat4, pY693) AF488 (BD Biosciences), and IgG2a isotype control AF488 (BD Biosciences).

Data acquisitions were performed on a MacsQuant (Miltenyi Biotec), and data were analyzed with FlowJo (BD Biosciences).

### Imaging Flow Cytometry

Untouched CD8T cells were enriched from the PBMC of healthy donors by magnetic depletion with the CD8 T Cell Isolation Kit (Miltenyi Biotec). Cells were stimulated for 15 min with 1000 IU/ml IFNα in FCS/ATB-supplemented FCS and washed. Surface staining was performed with anti-CD5 APC Vio770, CD8 VioBlue, and CD161 APC, followed by fixation and permeabilization as described for the phosphoflow experiments, and stained with either anti-pSTAT4 (Y693) AF488 or IgG2a Isotype control. Data were acquired on the Amnis Image Stream X Mark II and analyzed with IDEAS software (Merck Millipore).

### QRT-PCR

mRNA was extracted from isolated MAIT and memory CD8 T cells using the microRNA easy plus kit (Qiagen), according to the manufacturer’s instructions. Protein contamination was excluded by quantification of the sample absorbance at 260/280. mRNA samples were reverse transcribed with the SuperScript III First-Strand Synthesis System for RT-PCR (Thermo Fisher Scientific). QRT-PCR was performed with the SYBR Green Real-Time PCR Master Mix (Thermo Fisher Scientific), at a hybridation temperature of 58°C, on a LightCycler 480 (Roche). The following primers were used: STAT4 5′-GGCAATTGGAGAAACTAGAGG-3′ and 5′-AGGGTGGGTTGGCATACAT-3′; STAT1: 5′-TCACATTCA CATGGGTGGAG-3′ and 5′-CAAAGGCATGGTCTTTGTCA-3′; GAPDH: 5′-ATCTTCTTTTGCGTCGCCAG-3′ and 5′-ACGACCAAATCCGTTGACTCC-3′.

### Microarray Analysis

Gene expression analysis was performed on paired purified MAIT and conventional memory CD8 T cells from healthy donors (*n* = 4), either unstimulated or stimulated with 2 000 IU/ml of IFNα for 90 min at the GeT facility (INSA, Toulouse, France). RNA was extracted with microRNA easy plus kit (Qiagen), according to the manufacturer’s instructions. The quality of RNA was determined with an automated electrophoresis tool (Agilent Technologies, 2100 Bioanalyzer system) and the RNA quantity with a UV–Vis spectrophotometer (Thermo, NanoDrop 2100) at 260 nm/280 nm/230 nm absorbance. 500 pg of total RNA was used for sample preparation with the GeneChip Pico Reagent Kit. Following fragmentation, 5.5 μg of cRNA was hybridized for 16 hr at 45°C on human GeneChip Clariom S Array. GeneChips were washed and stained in the Affymetrix Fluidics Station 450. Then, GeneChips were scanned using the GeneArray Scanner 3000 7G and images were analyzed using Command Console software to obtain the CEL files with raw data (values of fluorescent intensity). Microarray analysis was carried out by the Transcriptome Analysis Console (TAC, version 4.0) software certified by Affymetrix. Raw data were transformed in log2, and Affymetrix Gene microarrays were normalized with the “Signal Space Transformation-Robust Multichip Analysis” (SST-RMA) method. After summarization of probes, a unique value for each gene was obtained. To remove false positives, the false discovery rate correction was applied with a *p*-value-adjusted threshold of 0.05.

### Statistical Analysis

Statistical analyses were performed with the GraphPad Prism 6.0 software. Paired or unpaired, two-tailed, and non-parametric Mann–Whitney tests were used to assess statistical significance. A *p*-value < 0.05 was considered significant.

## Results

### Role of Type 1 IFN in the Adjuvant Effect of TLR Agonists on CD161^hi^CD8^+^ T Cells

**FIGURE 1:**
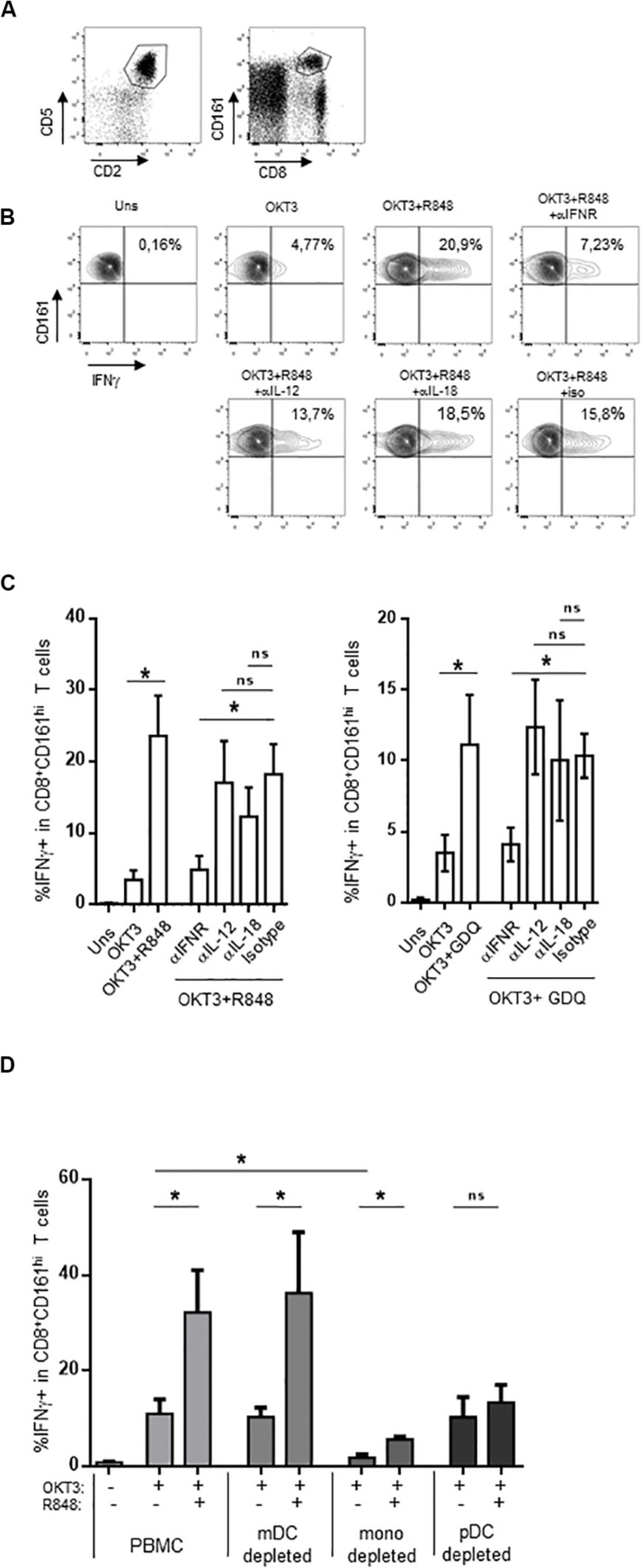
CD161^hi^CD8^+^ T cell activation by TLR7/8 ligands. **(A)** Gating strategy, based on T cell gating as CD5 + CD2 + lymphocytes (left panel), followed by identification as CD161^hi^CD8^+^ cells within the CD5 + CD2 + subset. **(B,C)** PBMC were rested or incubated for 16 h with the indicated reagents, in the presence of brefeldin A, before cell surface staining, fixation, permeabilization, and intracellular staining with anti-IFNγ antibody. CD161^hi^CD8^+^ T cells were identified as described in **(A)**. **(B)** A representative example obtained with R848 in one donor. **(C)** The cumulative frequencies (mean ± SEM) of IFNγ-producing CD161hi CD8 + T cells when R848 (left panel) or GDQ (right panel) was used as a co-stimulator, *N* = 4. **(D)** PBMC were used unseparated or after depletion of CD11c-, CD14-, or CD123-expressing cells, and stimulated with OKT3 in the presence or absence of R848. After staining, CD161hi CD8 + T cells were analyzed for intracellular IFNγ production. Cumulative frequencies (mean ± SEM) of IFNγ + MAIT cells are shown, *N* = 4. Mann–Whitney test was used to compare frequencies. *p < 0.05; ns, not significant.

Compared to conventional memory T cells, MAIT cells are hypofunctional to TCR-dependent stimulation, resulting in a blunted functional response to purified antigens or anti-CD3 stimulation (37, 38). A strong response can be obtained by the use of adjuvants such as TLR ligands. In particular, the TLR7/8 ligand R848 strongly activates monocytes, which in turn secrete IL-12 and IL-18; these two cytokines strongly potentiate TCR-dependent MAIT cells response. We reasoned that other signals could be involved in this indirect effect of R848; in particular, R848 binds TLR8 but also TLR7, which is strongly expressed by IFNα-secreting plasmacytoid dendritic cells (51–54). We thought that type 1 interferons could also be involved in the potentiation of MAIT cell activation, and tested this hypothesis in whole PBMC. We stimulated PBMC with a low dose of soluble OKT3 (sOKT3), with or without a previous incubation with R848, and quantified the frequency of MAIT cells expressing intracellular IFNγ. Anti-CD3 stimulation induces CD3 downregulation and may lead to experimental artifacts (55), precluding the use of both anti-CD3 and anti-TCR mAbs. It is well known that MAIT cells comprise >90% of CD8^+^CD161^hi^ T cells (56). Thus, we used the CD5^+^CD2^+^CD8^+^CD161^hi^ phenotype to study a CD8^+^ MAIT cell-enriched subset (Figure 1A). OKT3 stimulation alone resulted in a low proportion of IFNγ+CD161^hi^CD8^+^ T cells (Figures 1B,C), whereas the presence of R848 dramatically increased this functional response, both with respect to the frequency of IFNγ-producing cells and the amount of IFNγ/per cell (assessed by the MFI of IFNγ staining). We then tested the role of IL-12, IL-18, and type 1 IFN by testing the ability of blocking antibodies against these cytokines or their receptors to inhibit the action of R848. IL-12 and IL-18 blockade showed a minor effect on R848-induced potentiation of MAIT cell response (Figures 1B,C, left panel). In contrast, blocking the common IFNα/β receptor (IFNAR) dramatically inhibited the effect of R848 on IFNγ production in anti-CD3-stimulated CD161^hi^CD8^+^ T cells, suggesting a major role for type I IFN in this adjuvant effect. To confirm these data, we made use of another TLR agonist, gardiquimod (GDQ). GDQ is a specific TLR7 agonist and potent IFNα inducer. As shown in Figure 1C (right panel), GDQ significantly potentiated the CD161^hi^CD8^+^ T cell response to sOKT3, and this effect was reversed by blocking the IFNAR. These two pieces of data confirm that type 1 IFN is strongly involved in the potentiation of CD161^hi^CD8^+^ T cell response upon TLR7 (GDQ) or TLR7+8 (R848) ligation. pDC are the major IFNα-producing cells (on a per cell basis) upon TLR7 activation, but TLR8 agonists may also induce a strong production of IFNβ by monocytes or mDC (57). To identify the main cellular source(s) of type 1 IFN in our settings, we performed cell-depletion experiments. Thus, we separately depleted mDC, monocytes, and pDC from the PBMC of 3 individuals and assessed the effect of R848 on CD161^hi^CD8^+^ T cell response to anti-CD3 stimulation (Figure 1D). The depletion of mDC had no impact on CD161^hi^CD8^+^ T cell response to sOKT3 alone or with R848, suggesting that R848 can mediate its effects in the absence of mDC. Depleting monocytes resulted in a very low response to sOKT3, probably because this accessory cell type is necessary to the stimulatory effect of sOKT3 on T cells in the absence of exogenous co-stimulation. Nevertheless, the addition of R848 increased the CD161^hi^CD8^+^ T cell response to the same extent as in whole PBMC, compared with OKT3 alone. Finally, depleting pDC completely abolished the adjuvant effect of R848, strongly pointing to this cell type as the main mediator of R848 action on CD161^hi^CD8^+^ T cells. Altogether, these data show that R848 potentiates CD3-mediated CD161^hi^CD8^+^ T cell response in a pDC- and type 1 IFN-dependent manner. Since GDQ, a TLR7 agonist, shows similar adjuvant effects on CD161^hi^CD8^+^ T cells, it seems likely that R848 activates pDC through TLR7 as well in this setting. Therefore, we decided to analyze in depth the co-stimulatory action of type 1 IFN on MAIT cells response, selecting recombinant IFNα2b as a model type 1 IFN.

### IFNα Strongly Potentiates CD161^hi^CD8^+^ T Cells Effector Functions

**FIGURE 2:**
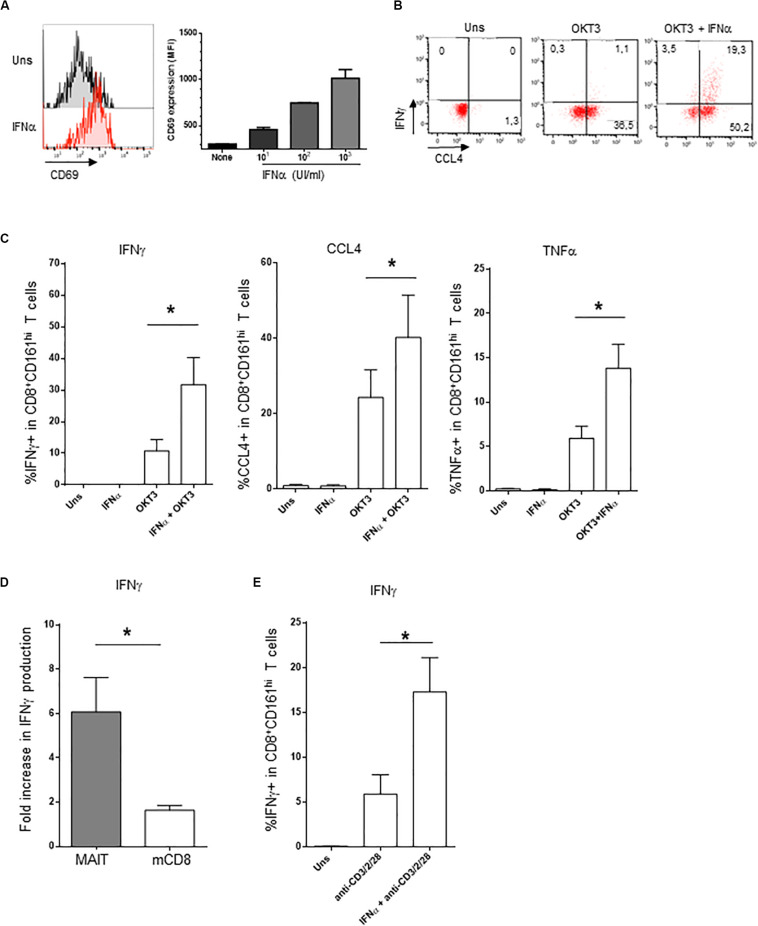
Co-stimulation of CD161^hi^CD8^+^ T cells activation by IFNα2b. **(A)** Left panel: representative example of membrane CD69 expression by CD161^hi^CD8^+^ T cells left unstimulated (upper panel) or stimulated for 20 h with 1000 IU/ml of rIFNα2b (lower). Right panel: dose-response of CD69 expression by CD161^hi^CD8^+^ T cells with increasing doses of rIFNα2b. **(B)** Representative example of intracellular IFNγ and CCL4 production by CD161^hi^CD8^+^ T cells left unstimulated or after stimulation with the indicated stimuli. **(C)** Cumulative frequencies (mean ± SEM) of intracellular IFNγ + (left), CCL4+ (middle), and TNFα + (right) CD161^hi^CD8^+^ T cells treated as in **(B)**, *n* = 6. **(D)** Ratio (mean ± SEM) of IFNγ + cells after OKT3 + IFNα2b stimulation to OKT3 stimulation alone, in CD161^hi^CD8^+^ T and conventional memory CD8 T cells. **(E)** Intracellular IFNγ production (mean ± SEM) by purified MAIT cells left unstimulated or stimulated with anti-CD3/CD2/CD28 tetrameric antibodies alone or in combination with IFNα2b, *n* = 4. Mann–Whitney test was used to compare frequencies. *p < 0.05; ns, not significant.

The addition of IFNα alone induced CD69 expression on PBMC CD161^hi^CD8^+^ cells in a dose-dependent manner (Figure 2A); however, the production of intracellular IFNγ was minimal (Figures 2B,C). In contrast, IFNα very strongly potentiated the production of IFNγ and TNFα, but also of CCL4, by CD161^hi^CD8^+^ T cells in response to anti-CD3 (Figures 2B,C). Type 1 IFN have pleiotropic effects, as most lymphocytes express the IFNAR. Therefore, the observed action of IFNα on CD161^hi^CD8^+^ T cells could only be representative of its broad action on memory CD8 T cells in general. To test this, we analyzed within the same PBMC the effect of IFNα on CD161^hi^CD8^+^ T cells and conventional CD8 memory T cells (as defined by the CD5^+^CD8^+^CD45RA^−^CD161^−^ phenotype). This adjuvant effect of IFNα on cytokine production was dramatically higher in CD161^hi^CD8^+^ T cells than in conventional memory CD8 T cells (Figure 2D). Thus, although IFNα acts on conventional memory CD8 T cells, CD161^hi^CD8^+^ T cells, including MAIT cells, display a higher sensitivity to this cytokine.

As our experiments were performed on whole PBMC, IFNα could act either directly on CD161^hi^CD8^+^ T cells or indirectly, by inducing in accessory cells other soluble or membrane factors that would co-stimulate this subset. To address this question, we purified MAIT cells (this time using anti-TCRVα7.2 antibody, see section “Materials and Methods”) and stimulated them with tetrameric CD2/CD3/CD28 antibodies alone or in the presence of IFNα. As shown in Figure 2E, purified MAIT cells responded directly to IFNα co-stimulation, in the absence of accessory cells (data not shown). Similar results were obtained with purified CD8 + T cells. We conclude that MAIT cells respond directly to type 1 IFN co-stimulation which synergizes with TCR stimulation for IFNγ production.

**FIGURE 3:**
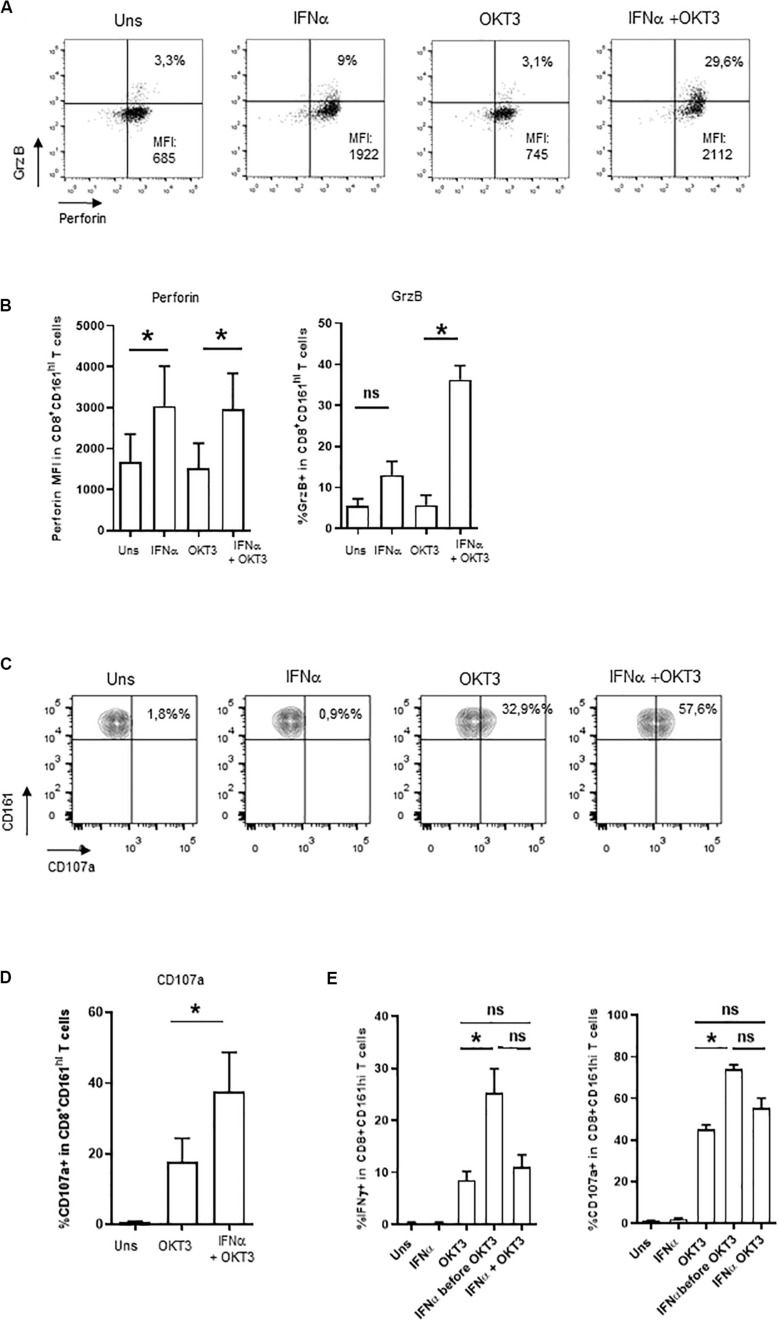
Induction of cytotoxicity in IFNα2b-co-stimulated CD161^hi^CD8^+^ T cells. **(A)** Representative example of intracellular perforin and granzyme B expression by CD161^hi^CD8^+^ T cells left unstimulated or stimulated under the specified conditions. **(B)** Cumulative data (mean ± SEM) for the mean fluorescence intensities of perforin staining (left panel) and frequencies of granzyme B + (right panel) CD161^hi^CD8^+^ T cells, stimulated in the indicated conditions. **(C)** Representative example of CD107a staining by CD161^hi^CD8^+^ T cells. Dot plots are gated on CD8^+^ T cells. **(D)** Cumulative frequencies (mean ± SEM) of CD107a^+^CD161^hi^CD8^+^ T cells after stimulation with OKT3 alone or in combination with IFNα2b. Mann–Whitney test was used to compare frequencies. *n* = 4, *p < 0.05; ns, not significant. **(E)** PBMC were either left unstimulated, or stimulated in different conditions: IFNα or OKT3 for 20 h, IFNα for 3 h before adding OKT3 for the next 17 h, or IFNα and OKT3 for 20 h. Brefeldin A was added 4 h after the beginning of the culture. At the end of the incubation period, PBMC were collected and stained for extracellular and intracellular markers as stated in the section “Materials and Methods.” For CD107a expression, the anti-CD107a mAb was incubated with cells during the whole culture period. Figures show cumulative data (*n* = 4) for IFNγ production (left panel) and CD107a expression (right panel) by CD161^hi^CD8^+^ T cells under the specified conditions. Statistical comparison was performed with a Friedman test with Dunn’s correction for multiple comparisons. *p < 0.05; ns, not significant.

Besides cytokine production, MAIT cells display cytotoxic functions; however, resting MAIT cells express low levels of cytolytic molecules and require some level of priming to display their full effector capacities (12, 13). We then asked whether type 1 IFN may prime CD161^hi^CD8^+^ T cells for cytotoxicity. IFNα alone was able to increase both perforin and granzyme B expression in CD161^hi^CD8^+^ T cells (Figures 3A,B), whereas OKT3 had no effect on these 2 molecules. When looking at the combined effects of these 2 stimuli, the level of perforin expression remained unchanged as it reached the same level than IFNα alone, but there was a very strong potentiation of granzyme B expression. We also tested the capacity of IFNα-co-stimulated CD161^hi^CD8^+^ T cells to degranulate, as assessed by the surface expression of the endosomal marker CD107a. IFNα alone did not induce CD107a expression, but it dramatically increased its expression upon sOKT3 stimulation (Figures 3C,D). Altogether, IFNα very strongly potentiates CD161^hi^CD8^+^ T cell effector functions, in terms of cytokine production, degranulation, and expression of cytotoxic molecules. It is noteworthy that the effect of IFNα was seen only when it was added before sOKT3, but not at the same time (Figure 3E). This suggests that IFNα acts by priming MAIT cells for full response to TCR-dependent stimulation.

**FIGURE 4:**
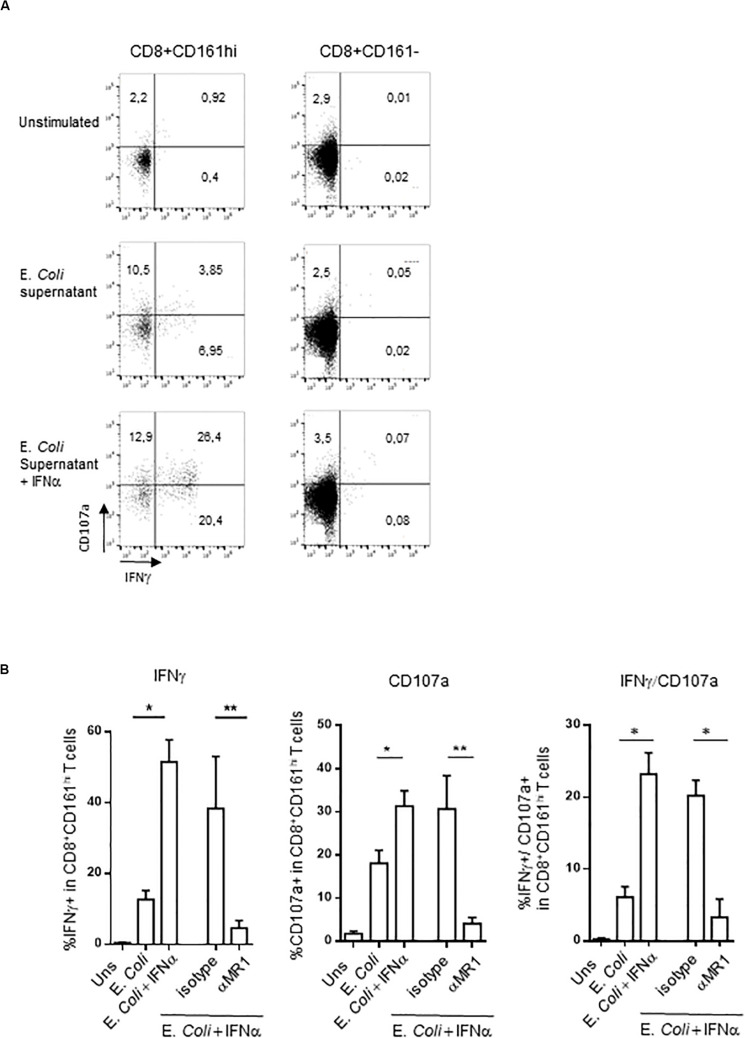
**(A)** Representative flow cytometry dot plots showing membrane CD107a and intracellular IFNγ expression by CD161^hi^CD8^+^ T cells (left) or conventional CD8 T cells (right) left unstimulated, or after stimulation with *E. coli* supernatant alone or in combination with IFNα2b. **(B)** Cumulative frequencies (mean ± SEM) of IFNγ+ (upper) and CD107a+ (lower panel) CD161^hi^CD8^+^ T cells stimulated under the indicated conditions, *N* = 5. Mann–Whitney test was used to compare frequencies. **p < 0.01, *p < 0.05; ns, not significant.

We reproduced these data by using a different, more specific stimulus for MAIT cells. We used the *E. coli* supernatant, which contains bioactive MR1 ligands (58). This supernatant is able to induce both intracellular IFNγ production and surface expression of CD107a in PBMC CD161^hi^CD8^+^ T cells (Figures 4A,B), in an MR1-dependent manner (Figure 4B). In contrast, the frequency of responding CD161^−^CD8^+^ T cells was very low. It is noteworthy that IFNα pretreatment especially increased the frequency of polyfunctional CD107a^+^/IFNγ^+^ MAIT cells (Figures 4A,B, right panel).

### Preferential Use of STAT4 in IFNα-Stimulated MAIT Cells

**FIGURE 5:**
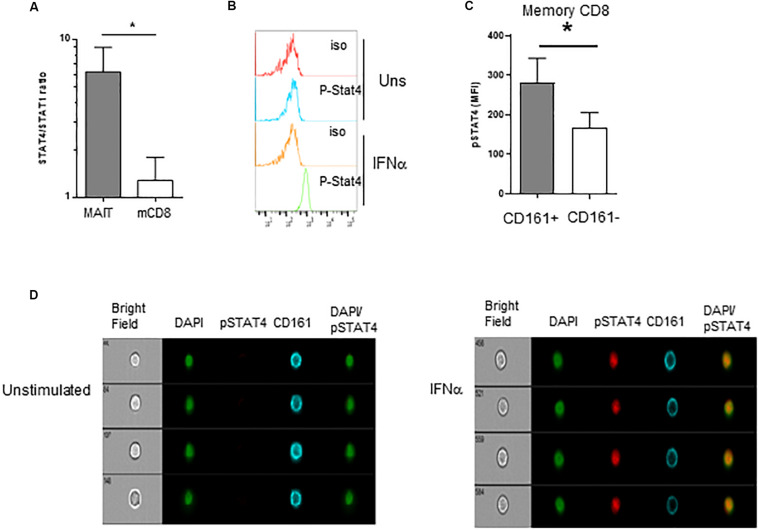
Preferential usage of the STAT4 pathway by IFNα-stimulated MAIT cells. **(A)** Ratio of STAT4 to STAT1 mRNA expression in purified MAIT cells and conventional memory CD8 T cells, determined by quantitative RT-PCR, *N* = 4. **(B)** Representative flow cytometry histograms of phospho-stat4 expression by MAIT cells left unstimulated or after IFNα2b stimulation. **(C)** Cumulative data (mean ± SEM) of MFI of pSTAT4 staining in MAIT and conventional memory CD8 T cells, after IFNα2b stimulation. Mann–Whitney test was used to compare frequencies. *n* = 6; *p < 0.05. **(D)** Representative example of flow imaging data, showing pSTAT4 and CD161 expression in CD161^+^CD8^+^ MAIT cells, left unstimulated (left panels) or after IFNα2b stimulation (right panels).

We then wanted to investigate what appears to be a specific sensitivity of MAIT cells to IFNα. Type 1 IFN have pleiotropic effects on different cell types; moreover, depending on the context, it can even show opposite effects on the same cell types. For example, in CD8 T cells, type 1 IFN can mediate either anti-proliferative effects or promote IFNγ production, akin to IL-12 (59–61). The mechanisms behind these differential effects have been partially unraveled in mice. It was shown in the murine LCMV model that the balanced expression of STAT4 and STAT1 modulates the response of CD8 T cells and NK cells to type 1 IFN. Indeed, although type 1 IFN predominantly uses the STAT1 pathway, leading to anti-proliferative effects, viral infection induces predominant STAT4 expression and promotes IFNγ (62). From these data, we hypothesized that the response of MAIT cells to IFNα could be at least partially explained by the differential use of STAT4 and STAT1-mediated pathways downstream of the IFNAR. To test this hypothesis, we first quantified STAT1 and STAT4 gene expression by QRT-PCR in purified MAIT (defined with the anti-TCR Vα7.2 mAb, see section “Materials and Methods”) versus TCRVα7.2-CD161- memory CD8 T cells. As shown in Figure 5A, the STAT4/STAT1 ratio in MAIT cells was >6 fold higher than in TCRVα7.2^−^CD161^−^CD8^+^ T cells, showing that resting MAIT cells chiefly express STAT4 over STAT1. To verify that STAT4 is indeed phosphorylated upon IFNα incubation, we used phosphoflow cytometry to quantify p-STAT4. STAT4 was phosphorylated upon 15 min of incubation with IFNα in both MAIT and conventional memory CD8 T cells (Figure 5B); however, the MFI of pSTAT4 expression was significantly greater in the former than in the latter (Figure 5C). Finally, we checked the nuclear translocation of p-STAT4 in MAIT cells by use of imaging flow cytometry. Resting, inactivated MAIT cells (defined as CD161^+^CD8^+^ cells) expressed no p-STAT4; upon 15 min of incubation with IFNα, we could detect a significant expression of pSTAT4, which entirely co-localized with DAPI in the nucleus of MAIT cells (Figure 5D). We conclude from all this set of experiments that resting MAIT cells express an elevated level of STAT4, which is preferentially phosphorylated and translocated in the nucleus upon IFNα incubation, compared to conventional memory CD8 T cells.

### IFNα Elicits a Specific Transcriptional Program in MAIT Cells

**FIGURE 6:**
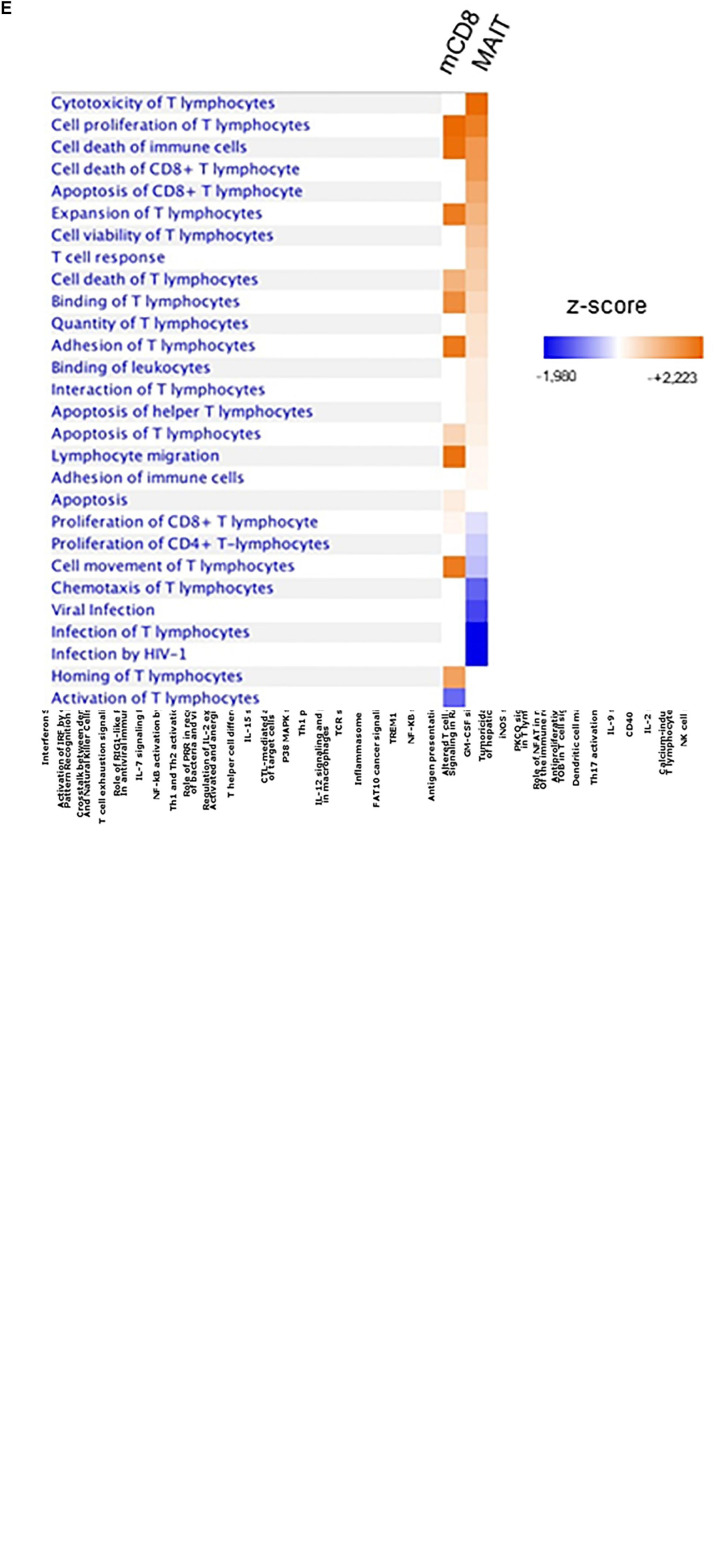
IFNα2b induces a specific set of genes in MAIT cells. **(A)** Principal component analysis of gene expression by untreated MAIT cells (blue), IFNα-stimulated MAIT cells (red), conventional memory CD8^+^CD161^–^ T cells (purple), and IFNα-stimulated conventional memory CD8^+^CD161^–^ T cells (green). Analysis was performed with the Transcriptome Analysis Console (TAC, version 4.0) software. **(B)** Volcano plot of differentially expressed genes (DEG) between MAIT and conventional memory CD8 T cells. Colored dots represent genes with at least a 2-fold difference. The main MAIT-associated genes are labeled. **(C)** Venn diagrams of the number of DEG induced by IFNα2b stimulation in conventional CD8 memory and MAIT cells. **(D)** Pathway analysis of the genes differentially expressed in IFNα2b-stimulated versus unstimulated MAIT cells. The left axis represents the % of genes within each pathway, and the right axis shows the –log (*p*-value). Red bars represent upregulated, and green bars downregulated genes. Orange dots and line show the –log (*p*-value) for each pathway. **(E)** Comparison of the main pathways regulated by IFNα2b stimulation in conventional memory CD8 versus MAIT cells. Four different donors were analyzed in these experiments. Pathway analyses were performed with the Ingenuity Pathway Analysis software.

Our data strongly suggest that IFNα selectively acts on MAIT cells to dramatically increase their responsiveness to TCR-dependent activation. To more formally address this hypothesis, we performed a microarray analysis of gene expression regulated by IFNα in MAIT versus conventional memory CD8 T cells. Of note, the isolation procedure excluded TCRVα7.2^−^CD161^−^ cells from this latter subset. MAIT and conventional memory CD8 T cells were purified from the blood of 4 healthy donors, and whole-genome microarrays were performed on resting and IFNα-stimulated samples. We deliberately chose an early time-point (90 min after stimulation) to investigate the immediate effect of type 1 IFN on gene expression. PCA analysis of the 4 types of samples confirmed that resting MAIT cells express a specific set of genes (Figure 6A). We first looked at the expression of genes that have been previously described as MAIT-enriched. Indeed, we found a high expression (compared to conventional memory CD8) of *KLRB1* (encoding CD161), *ZBTB16* (encoding PLZF), *CCR6*, *RORC*, *IL-23R*, *CXCR6*, *DPP4* (encoding CD26), and *IL18R1* (Figure 6B). These data confirmed the quality of our protocol and encouraged us to analyze further comparisons. A total of 610 and 928 genes were found to be significantly regulated by IFNα in conventional memory CD8 and MAIT cells, respectively (Figure 6C). Interestingly, 464 genes were found in common between both cell types, including multiple interferon-specific genes (ISG) as expected (Supplementary Table S1). Half (464) of the genes regulated in MAIT were specific and not found in conventional memory T cells. We took a closer look and found that IFNα downregulated 189 genes in conventional CD8 and 430 genes in MAIT, 70% of the latter being specific (Figure 6C, right panel, middle). In contrast, IFNα upregulated 421 genes in conventional CD8 and 498 genes in MAIT cells, with only 33% of the latter being specific to this subset (Figure 6C, left panel). In other words, IFNα down- and upregulates approximately the same number of genes in MAIT cells, whereas more genes are upregulated than downregulated in conventional CD8 T cells. Interestingly, *furin*, encoding a proprotein convertase shown to be involved in IFNγ production (63), showed an increased transcription in treated MAIT cells only, providing a possible mechanism for the selective action of type 1 IFN. We then used the Ingenuity Pathway Analysis software to analyze canonical pathways regulated by IFNα in MAIT cells. As expected, we obtained very low *p*-values for pathways directly associated with IFN signaling, interferon-regulatory factors, or pattern-recognition receptors (Figure 6D). Interestingly, we also detected modules associated with cytotoxic lymphocytes and natural killer cell signaling. We next analyzed side-by-side the disease and function pathways regulated by IFNα in conventional CD8m and MAIT cells. This revealed common and specific pathways for both subsets. Interestingly, MAIT cells specifically upregulated pathways associated with cytotoxicity of lymphocytes, and T cell response, compared with conventional CD8m T cells (Figure 6E). Altogether, these experiments confirm that the MAIT cell response to IFNα partially induces a specific set of genes, not found in conventional memory CD8 T cells, and therefore that MAIT cells display a specific responsiveness to this cytokine.

## Discussion

How pathogen-derived signals activate protective antimicrobial functions in MAIT cells remains incompletely solved. We provide here strong evidences that IFNα2b (and most likely other type 1 IFN) are important actors in this process, confirming a very recent report (64). We also provide compelling evidences that IFNα induce a specific signaling and transcriptional program in MAIT cells, which could be harnessed for future intervention.

Type 1 IFN have broad actions on the immune system, and one could argue that their stimulatory effects on MAIT cells only reflect their general action on CD8 T cells. However, this is actually not the case (65). In *in vivo* murine models, IFNα mostly stimulates naïve CD8 T cells and fosters their differentiation into memory cells (66, 67). On the contrary, its effect on memory CD8 T cells is mostly anti-proliferative. Similarly, IFNα promotes memory differentiation from human naïve CD8 T cells *in vitro* (68) and only modestly increases cytokine production by CD8m T cells (69). In contrast, we showed that MAIT cells responded promptly to IFNα incubation by directly upregulating cytotoxic molecules and dramatically increasing cytokine response to TCR triggering. Although we did not directly address mechanistic issues, we provided circumstantial evidences that a preferential usage of the Stat4 pathways by the IFNAR signaling is occurring in MAIT cells. It is well known that besides the canonical Stat1/Stat2 pathway, IFNAR signaling may involve any other Stats, depending on the cell type and probably other factors. Seminal work from the Biron laboratory has shown in the murine LCMV infection model that Stat4 is required for innate IFNγ production by natural killer cells but also that viral infection induces a switch in CD8 + T cells from a Stat1-dependent, anti-proliferative effect to a Stat4-dependent, IFNγ production in response to IFNα (62, 70, 71). Our data suggest that mature MAIT cells are equipped with a Stat4-dependent signaling module that drives their response to IFNα. If so, it remains to be determined when, how, and where this specific signaling pathway is plugged into the IFNAR receptor in MAIT cells. Thus, it is possible that thymic positive selection drives this process, perhaps upon PLZF expression, implying that MAIT cells would be developmentally programmed to provide this kind of response. Interestingly, type 1 IFN drives the development of innate-like CD8 T cells in mice (72). More studies are needed to address this question.

We found that IFNα has a potent effect when pre-incubated with PBMC before OKT3 stimulation. This is at odds with the report from Lamichhane et al. (64), where simultaneous treatment with IFNα and 5-A-RU/MG (a potent MAIT cell-stimulating ligand) showed a synergistic effect. The reason for this discrepancy is not clear but may involve the different setup between our two studies. Indeed, the use of 1 nM of 5-A-RU/MG is known to strongly activate MAIT cells through MR1 presentation by APC, whereas we deliberately chose to use a low dose of soluble OKT3. Our gene expression analysis confirmed that IFNα induces a partially specific transcriptional program, in line with MAIT cells’ specific sensitivity to IFNα. We chose a timeframe of 90 min to minimize positive and negative feedback loops that may occur in response to IFN; further, it is close to the peak of the IFN response as shown recently in human and mouse cells (73). We made the surprising finding that IFNα downregulates many more genes in MAIT cells than in conventional mCD8 T cells; indeed, it has been shown recently that in most leucocytes, more genes are upregulated than downregulated in response to IFNα signaling (73). Among these downregulated genes, we found genes involved in the regulation of TCR and NF-κB signaling (Supplementary Table S1). These data will require experimental validation. However, we speculate that the hypo-responsiveness of MAIT cells might be the consequence of the wiring of negative regulators of TCR signaling, which would then be downregulated by IFNα (and possible other signals), allowing full responsiveness to antigenic stimulation. If so, it will also raise the issue of the developmental regulation of this hypo-responsive phenotype in MAIT cells, from the thymus to the periphery.

Although the STAT4 pathway did not come out as strongly induced in MAIT cells in the microarray analysis, we found a selective induction of transcripts for *furin* in MAIT cells only. Furin is a ubiquitously expressed proprotein convertase with many substrates, involved in various biological processes. Interestingly, furin is induced by IL-12 in a STAT4-dependent manner and preferentially expressed in human Th1 cells (63). Another publication confirmed that *furin* is a target of STAT4 binding in Th1 cells (74). The absence of *furin* leads to a decreased production of IFNγ in Th1 cells, suggesting an IFNγ-enhancing role for this protease (63). It is therefore possible that furin expression is upregulated by IFNα in a STAT4-dependent manner in MAIT cells and could be important for the enhanced IFNγ production. Future studies will address this possibility.

Our work focused on CD8^+^ MAIT cells, which represent the majority of MR1-restricted T cells. The reason behind this strategy was our goal to select a homogeneous population to compare with their mainstream counterparts, i.e., conventional CD161^−^ memory CD161^hi^CD8^+^ T cells. However, this excluded CD8^−^ MAIT cells, composed of a very small fraction of CD4^+^ and a significant population of CD4^−^CD8^−^ (DN) MAIT cells (7). Furthermore, DN and CD8^+^ MAIT cells are developmentally related but display distinct transcriptional and functional profiles, with the CD8^+^ subset expressing elevated levels of NK-related markers (NKG2A, NKG2D, and others), cytotoxic molecules (GrzB), and type 1 cytokines (IFNγ, TNFα), compared to DN MAIT cells (75). Thus, it is possible that DN MAIT cells behave differently than their CD8^+^ counterparts with respect to their response to IFNα. Future studies will undoubtedly address this question.

We must stress that comparisons between MAIT cells and conventional memory CD8 + T cells involved slightly different definitions of this latter subset, including total CD8^+^CD161^−^ (functional studies), CD8^+^TCRVα7.2^+^CD161^−^ (QRT-PCT experiments) or CD8^+^TCRVα7.2^−^CD161^−^ (microarray analysis). Published data showed several differences between TCRVα7.2^+^ and TCRVα7.2^−^ cells within the CD8^+^CD161^−^ subset, although this analysis was performed without selecting specifically for memory cells (76). Nevertheless, it is conceivable that some of these comparisons between MAIT cells and conventional CD8^+^ cells are biased because of subset definitions. On the other hand, the TCRVα7.2^−^ represent a very minor subset of CD161^−^CD8^+^ T cells, which should not behave so much differently than other CD161^−^ cells. Although this represents an objective limitation, we believe this does not impede conclusions drawn from our experiments.

Previous work has already shown how inflammatory signals derived from TLR-stimulated APC synergize with TCR signaling to induce full effector functions in MAIT cells. Monocytes have been the main focus of these studies and are shown to be strong MAIT cells stimulators upon TLR8 or TLR4 stimulation (77). Of note, the specific soluble factors produced by monocytes and responsible for MAIT cells co-stimulation were not identified (39). In mice, *in vivo* activation of MAIT cells was observed only when TLR agonists (of TLR2, TLR3, or TLR9) were co-administered with the purified MR1 ligand 5-OP-RU. The specific APC(s) and cytokines involved in this effect were not investigated (37). We show here that pDC can be the source of a strong MAIT cell co-stimulation, at least upon TLR7 stimulation. pDC express MR1 transcripts and could be potent APC for MAIT cells. It remains to be investigated whether pDC are mostly (or solely) cytokine producers in this setting or are also able to potently present MR1 ligands to MAIT cells.

With regard to the prospect of harnessing MAIT cells’ functions in a therapeutic or prophylactic manner, a number of reports have demonstrated the importance of inflammatory cytokines in MAIT cell co-stimulation. Several authors have shown the potency of the IL-12 + IL-18 combination, where the role of IL-12 is probably to increase IL-18 responsiveness; on the other hand, IL-7 is also a potent MAIT cell co-stimulator but mostly for cytotoxicity more than for IFNγ production. Recent reports have provided insights into the effects of cytokine stimulation on MAIT cells at the transcriptional and functional levels, mostly focusing on IL-12 + IL-18 (41, 42, 44). These cytokines, along with others such as IL-15 and TL1-A, strongly synergize with TCR stimulation to induce full antimicrobial effector functions, including IFNγ production and cytotoxicity, akin to our observations with IFNα. In one paper, IL-12 + IL-18 stimulation alone was sufficient to induce the expression of IFNγ and cytotoxic effectors at the mRNA and protein levels. Further, most DEGs were upregulated in these studies, whereas we found a lot of downregulated genes in response to IFNα stimulation. It must be noted that these responses were described as slow, and analyzed at late time points (after 24 h of stimulation), suggesting that it could involve feed-forward loops that we tried to avoid in our study of the immediate response to IFNα. As we and others did not compare all these cytokines in their ability to influence MAIT cell activation, it would be of great interest to analyze the transcriptional response of MAIT cells to these stimuli, as a way to stratify their interest in the context of immune intervention but also to analyze in deeper molecular details the response of MAIT cells.

Besides these important issues, our work raises hypotheses and possibilities. For instance, it is well known that viral infections are strong inducers of type 1 IFN. A recent report very elegantly showed that a localized antiviral vaccination induces an IFN response that rapidly spreads to the entire organism, endowing distant cells from prophylactic antiviral mechanisms (78). It is tempting to suggest that this wave of IFN signaling would also provide a priming signal to circulating MAIT cells, which would then be ready for response. Viral infections often dampen inflammatory neutrophilic response, with increased susceptibility to bacterial infections. Priming this specific, highly potent antibacterial subset of T cells would make high evolutionary sense to avoid major damages due to secondary infections. More generally, the type 1 IFN response in antibacterial and fungal immunity is often assumed to be detrimental (79), but this is still debated. Our data emphasize, at least from a MAIT-centered prospective, that IFNα can be beneficial for antibacterial mechanisms and may foster new endeavors to analyze the therapeutic and prophylactic consequences.

## Data Availability Statement

The raw data supporting the conclusions of this article will be made available by the authors, without undue reservation, to any qualified researcher.

## Ethics Statement

Ethical review and approval was not required for the study on human participants in accordance with the Local Legislation and Institutional Requirements. Written informed consent for participation was not required for this study in accordance with the National Legislation and the Institutional Requirements.

## Author Contributions

MP, CG, and CC performed the experiments and analyzed the data. TS helped with preparation of *E. coli*. ET designed the study, analyzed the data, and wrote the manuscript. All authors contributed to the article and approved the submitted version.

## Conflict of Interest

The authors declare that the research was conducted in the absence of any commercial or financial relationships that could be construed as a potential conflict of interest.
